# **α**,β-Dehydroamino acids in naturally occurring peptides

**DOI:** 10.1007/s00726-014-1846-4

**Published:** 2014-10-17

**Authors:** Dawid Siodłak

**Affiliations:** Faculty of Chemistry, University of Opole, Oleska, 48 45-052 Opole, Poland

**Keywords:** Dehydroamino acids, Dehydropeptides, Natural products, Z/E isomerisation, Methylation, Depsipeptides, Heterocycles

## Abstract

α,β-Dehydroamino acids are naturally occurring non-coded amino acids, found primarily in peptides. The review focuses on the type of α,β-dehydroamino acids, the structure of dehydropeptides, the source of their origin and bioactivity. Dehydropeptides are isolated primarily from bacteria and less often from fungi, marine invertebrates or even higher plants. They reveal mainly antibiotic, antifungal, antitumour, and phytotoxic activity. More than 60 different structures were classified, which often cover broad families of peptides. 37 different structural units containing the α,β-dehydroamino acid residues were shown including various side chains, *Z* and *E* isomers, and main modifications: methylation of peptide bond as well as the introduction of ester group and heterocycle ring. The collected data show the relation between the structure and bioactivity. This allows the activity of compounds, which were not studied in this field, but which belong to a larger peptide family to be predicted. A few examples show that the type of the geometrical isomer of the α,β-dehydroamino acid residue can be important or even crucial for biological activity.

**Electronic supplementary material:**

The online version of this article (doi:10.1007/s00726-014-1846-4) contains supplementary material, which is available to authorized users.

## Introduction

**Scheme 1:**
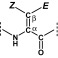
General scheme of α,β-dehydroamino acid residue

Dehydroamino acids belong to non-coded amino acids found in nature. Their main structural feature is carbon–carbon double bond, which in most cases is placed between the carbon atom α of the main chain and the carbon atom β of the side chain of the α-amino acid. Therefore, the term dehydroamino acids, although has a broader meaning, usually concerns α,β-didehydro-α-amino acids (Scheme 1), for convenience named short α,β-dehydroamino acids, and it will also be used in this work.

The α,β-double bond has a profound effect on the conformational properties of the dehydroamino acid residue and, in consequence, on the conformation of the whole peptide molecule. Due to planar hybridisation *sp2*, the carbon atom α does not have asymmetry, an important and characteristic of the standard amino acids. The bonds of the carbon atoms α and β are shorter, but bond angles are larger than in the saturated *sp3* analogues. The α,β-double bond constricts the topography of the side chain and limits the position of the β-substituents, which leads to the appearance of isomers Z and E. Furthermore, possible co-planarity of the α,β-double bond and flanking amide groups enable π-electron conjugation, which should be considered as a stabilising force of selected conformations. The α,β-double bond undergoes many reactions, particularly Michael addition, *Z/E* isomerisation, hydrogenation, and cycloaddition (Humphrey and Chamberlin 1997). Therefore, α,β-dehydroamino acids can play an effector role. Both conformational properties and chemical reactivity of the α,β-dehydroamino acids can influence the bioactivity of the dehydropeptides.

The conformational characteristics of peptides containing α,β-dehydroamino acids (Jain and Chauhan 1996; Mathur et al. 2003; Gupta and Chauhan 2010) as well as the methods of their synthesis (Humphrey and Chamberlin 1997; Bonauer et al. 2006) have been previously reviewed. The last general review concerning the α,β-dehydroamino acids appeared in 1983 (Noda et al. 1983). A considerable part of this review concerns piperazinediones as well as compounds, in which the α,β-dehydroamino acid fragments can be seen, but it is difficult to perceive them as independent structural units. Two bigger classes of natural compounds containing the α,β-dehydroamino acids, lantibiotics (Chatterjee et al. 2005; Bierbaum and Sahl 2009; Jack and Jung 2000; Dischinger et al. 2013), and thiopeptide antibiotic (Bagley et al. 2005; Just-Baringo et al. 2013) were also extensively studied.

Nevertheless, during the last two decades, numerous natural peptides containing the α,β-dehydroamino acids have been isolated and their structures determined. This review describes these findings. In particular, the work focuses on the peptides structure, structural similarities, occurrence of *Z* and *E* isomers of the α,β-dehydroamino acids, and the biological source and activity of dehydropeptides. For clarity, the literature data concerning dehydropeptides were collected according to the type of α,β-dehydroamino acid, and then by further main chain modifications.

### Dehydroamino acids

This part describes naturally occurring peptides that contain the α,β-dehydroamino acid residue(s), which can be simply derived from standard amino acids.

#### Dehydroalanine and dehydrobutyrine

Dehydroalanine (ΔAla) is the simplest dehydroamino acid with the shortest side chain constituted by the methylidene group. Thus, it does not reveal geometrical isomers. Contrarily, dehydrobutyrine (ΔAbu) is the simplest α,β-dehydroamino acid, which has isomers *Z*/*E*. The most stable isomer is the Z (Dugave and Demange 2003) and is thus present in a majority of compounds containing the ΔAbu residue.

The oldest known dehydropeptide is nisin **1**, produced by bacteria *Lactococcus lactis* and applied as a food preservative even presently (Mattick et al. 1947; Gross and Morell 1971). Nisin is the prototype of lantibiotics, a large family of about 80 compounds produced by Gram-positive bacteria and thus extensively reviewed (Chatterjee et al. 2005; Bierbaum and Sahl 2009; Jack and Jung 2000; Dischinger et al. 2013). The characteristic structural feature of lantibiotics is lanthionine or 3-methyllanthione. Usually, lantibiotics contain the ΔAla and (*Z*)-ΔAbu residues.

AM-toxins **2** are produced by fungi and are one of the oldest known types of dehydropeptides. These host-specific phytotoxins are produced by the pathogenic strain *Alternaria alternata*, thus formerly described as alternariolides, and cause leaf spot disease in apple trees (Okuno et al. 1974; Miyashita et al. 2003). This group of three cyclodepsitetrapeptides fully comprise ΔAla as well as other non-standard residues. The structural differences are located in a type of substituent at the phenyl ring (Ueno et al. 1975a, b, c).

Two marine ascidiands, *Didemnum cuculiferum* and *Polysyncranton lithostrotum*, are a source of vitilevuamide **3**, a bicyclic tridecadepsipeptide containing the ΔAla residue. Vitilevuamide reveals anticancer activity and is cytotoxic in several human tumour cell lines (Edler et al. 2002).

Corpeptin A **4** is produced by bacteria *Pseudomonas corrugate* and shows phytotoxic and antibacterial activity (Emanuele et al. 1998). This 22 amino acid lipodepsipeptide consists of a 17 amino acid linear chain connected with a cyclopentadepsipetide ring. The linear chain contains four α,β-dehydroamino acid residues, ΔAla and three ΔAbu, although the geometrical isomers of the latter were not determined.

Tolaasins **5** are pathogens produced by a virulent strain of Gram-negative soil bacteria *Pseudomonas tolaasii* (Nutkins et al. 1991; Rainey et al. 1991; Bassarello et al. 2004). There are 18 amino acid lipodepsipeptides, which contain the cyclopentadepsipetide ring and a 13 amino acid linear chain, in which two (*Z*)-ΔAbu residues are present, as confirmed by the NOE experiment. Tolaasins differ in the type of amino acid residue at position 15 and 16 as well as N-terminal acid. Tolaasin C is an acyclic form of tolaasin I.

Similar structures have fuscopeptins **6**, metabolites produced by *Pseudomonas fuscovaginae*, the causal agent of sheath brown rot of *Gramineae*. Fuscopeptins show antifungal activity and induce damage of plant tissue (Ballio et al. 1996; Corailola et al. 2008). These are 19 amino acid lipodepsipeptides, which consist of the cyclopentadepsipetide ring and a 14 amino acid linear chain, in which two (*Z*)-ΔAbu residues are present. Due to closely related structures as well as the bioactivity and the source of origin, the lipodepsipeptides **4-6** should be considered as a family.

Syringopeptins **7** and **8** are a family of other cyclic lipodepsipeptides produced by bacteria *Pseudomonas syringae*. They show phytotoxic as well as to some extent antifungal and antibacterial activity. Syringopeptin 25 (SP25) **7** consists of 25 amino acid residues; it is a cyclooctadepsipeptide ring connected with an 18 amino acid residue chain and contains four (*Z*)-ΔAbu residues (Ballio et al. 1991). There is also analogue [Phe^25^]SP25, in which phenylalanine is present instead of tyrosine (Scaloni et al. 1997).

Syringopeptins 22 (SP22) **8** consist of 22 amino acid residues: a cyclooctapeptide ring and a 14 amino acid chain, in which the N-terminal residue is *N*-acylated by a 3-hydroxylated fatty acid containing either 10 or 12 carbon atoms (SP22A and SP22B) (Ballio et al. 1991). In comparison to SP25, SP22s contain (*Z*)-ΔAbu instead of valine residue in the depsipeptide ring. There are also three other (*Z*)-ΔAbu residues in the peptide chain. Analogues were also found, such as syringopeptin Phv ([Leu^4^,Ala^7^]SP22) (Grgurina et al. 2005), syringopeptin SC ([Leu^4^,Leu^7^,ΔAla^9^]SP22), which contains one ΔAla residue (Isogai et al. 1995), syringopeptin 508 ([Leu^4^,Leu^7^,Ala^9^]SP22) with three (*Z*)-ΔAbu residues, and a 3-hydroxylated fatty acid containing either 12 or 14 carbon atoms (Grgurina et al. 2005). Due to similarities in structure and activity, the peptides **4-6,** and **7** and **8** are often compared.

Syringostatins **9** (Akira et al. 1990), like also syringotoxin (Ballio et al. 1990), syringomycins (Scaloni et al. 1994), pseudomycins (Ballio et al. 1994), and cormycin A (Scaloni et al. 2004) are a group of lipodepsipeptides with phytotoxic and antifungal activities, produced by bacteria *Pseudomonas*. The cyclic lactone ring consists of nine amino acid residues, including the (*Z*)-ΔAbu residue. The constant structural feature of these peptides is a fragment of four amino acids, (*Z*)-ΔAbu-l-Asp(3-OH)-l-Thr(4-Cl)-l-Ser with serine *N*-acylated by fatty acids of various lengths. These structural features, together with a similar source of origin and bioactivity mean that they can be regarded as a family of compounds.

Dolastatin 13 (sea hare *Dolabella auricularia*) (Pettit et al. 1989), symplostatin 2 (cyanobacteria *Symploca hydnoides*) (Harrigan et al. 1999), somamides (cyanobacteria *Schizothrix* and *Lyngbya majuscula*) (Nogle et al. 2001), lyngbyastatins 4-10 (cyanobacteria *Lyngbya confervoides* and *semiplena*) (Matthew et al. 2007; Taori et al. 2007; Kwan et al. 2009), bouillomides (cyanobacteria *Lyngbya bouillonii*) (Rubio et al. 2010), and molassamides B (cyanobacteria *Dichothrix utahensis*) (Gunasekera et al. 2010) can also be perceived as a family of compounds **10**. These cyclodepsipentapeptides contain (*Z*)-ΔAbu residue with C-terminal 3-amino-6-hydroxy-2-piperidone (Ahp). The main structural differences rely on short peptide chains at the N-terminus of threonine and on a substituent of the tyrosine ring. Although dolastatin 13, the first compound of this class, was obtained from sea hare, it is most probably of cyanobacterial origin as this shell-less mollusc has developed very powerful chemical defences by careful selection and/or biosynthetic manipulation of various dietary sources. The bioactivity of symplostatin 2 and somamides was not determined; nevertheless, the studies into dolastatin 13, lyngbyastatins 4-10, bouillomides, and molassamides show that these peptides are cell-growth inhibitors (selectively inhibit elastase and chymotrypsin). FR901277 **11** (Nakanishi et al. 1999) should be classified into the same family of compounds because of both structural and bioactive similarities (elastase inhibitor). Interestingly, it was isolated from bacteria *streptomyces resistomicificus*.

Loihichelins A-F **12** (Homann et al. 2009) are an example of linear dehydropeptides. This group of heptapeptides contains the (*Z*)-ΔAbu residue and C-terminal 3-amino-1-hydroxy-2-piperidone. Loihichelins differ in the type of N-terminal fatty acid. Isolated from culture of the marine bacterium *Halomonas* sp. LOB-5, heterotrophic Mn(II)-oxidising bacterium, and loihichelins are perceived as siderophores, playing a role in sequestering Fe(III) released during basaltic rock weathering. Their role in the promotion of Mn(II) and Fe(II) oxidation can also be considered.

Lavendomycin **13** has been isolated from culture filtrates of *Streptomyces lavendulae*. It exhibits a very low toxicity and a high antibiotic activity towards Gram-positive bacteria both in vivo and in vitro (Komori et al. 1985). Lavendomycin is a linear hexapeptide containing the (*Z*)-ΔAbu residue with stereochemical configuration assigned by total synthesis (Schmidt et al. 1990).

Stenothricin **14** is an inhibitor of bacterial cell wall synthesis isolated from *Streptomyces* (Hasenböhler et al. 1974). This antibiotic has a linear octapeptide structure containing the ΔAbu residue of unknown stereochemistry (Rinken et al. 1984).

FK228 (formerly named FR901228) **15** (Ueda et al. 1994) is an antitumour peptide produced by *Chromobacterium violaceum*. It was shown that reduction of an intramolecular disulphide bond of FK228 greatly enhances its inhibitory activity. Thus, FK228 serves as a stable prodrug to inhibit class I enzymes (potent histone deacetylase (HDAC) inhibitor) and is activated by a reduction after an uptake into the cells (Fumarai et al. 2002). This bicyclic pentadepsipeptide contains the (*Z*)-ΔAbu residue, the stereochemistry of which was confirmed both by NMR techniques and X-ray crystallography (Shigematsu et al. 1994).

Largamides A–C **16** (Plaza and Bewley 2006) are produced by marine cyanobacterium *Oscillatoria* sp. Their bioactivity has not been determined to date. Apart from the (*Z*)-ΔAbu residue, these pentadepsipeptides are characterised by the unusual occurrence of senecioic acid. Largamides B and C possess in addition the rare 2-amino-5-(4′-hydroxyphenyl)pentanoic and 2-amino-6-(4′-hydroxyphenyl)hexanoic acid, respectively.

Kahalalide F **17** (Hamann and Scheuer 1993; López-Macià et al. 2001) is a tridecapeptide isolated from a sacoglossan mollusc *Elysia rufescens*. The mollusc feeds on a green alga, *Bryopsis* sp., which is most probably the origin source. The bioassay results of antitumour, antiviral, antimalarial, and against AIDS OI pathogens are reported (Hamann et al. 1996). The antitumour activity is the most promising (García-Rocha et al. 1996). The (*Z*)-ΔAbu residue is placed in the depsipeptide ring. When the ester bond is hydrolysed, kahalalide F is transformed to kahalalide G, but the bioactivity is lost.

Stendomycins **18** (Bondaszky et al. Bodanszky et al. 1969) are antifungal antibiotics isolated from cultures of *Streptomyces endus*. Stendomycins contain a heptadepsipeptide ring and a heptapeptide linear chain with the ΔAbu residue and N-terminal fatty acid. The structure **18** represents the dominant compound of the stendomycin family. In other members of the stendomycin group, isomyristic acid is replaced by its lower homologues and alloisoleucine by valine or leucine. The configuration of the amino acid constituents was established by determination of the specific rotation of the amino acids isolated from an acid hydrolysate of stendomycin. However, the stereochemistry of the ΔAbu residue remains to be established.

*Erwinia herbicola* produces two acylated antibiotics, herbicolins A and B, **19** (Winkelmann et al. 1980; Aydin et al. 1985). The C-terminal arginine residue forms a lactone ring with the hydroxy group of l-threonine, while the N-terminus is acylated by the (*R*)-3-hydroxytetradecanoic acid residue. The ΔAbu residue is present in the side chain, but its geometric isomer remains unrecognised. Herbicolin A, the main component, has d-glucose moiety linked via a 1-α-glycosidic bond to the 3-hydroxytetradecanoic acid residue (Greiner et al. Greiner and Winkelmann 1991). Thus, herbicolin A was the first known glycosylated dehydropeptide. Herbicolin B is the aglycone of herbicolin A.

Another example of glycosylated lipopeptides are hassallidins **20** (Neuhof et al. 2005, 2006), with antifungal activity, from cyanobacterium *Hassallia* sp. Within these cyclic nonadepsipeptides five nonproteinogenic amino acids were found including the (*Z*)-ΔAbu residue, as confirmed by a homonuclear NOESY spectrum. The lipid part of the peptide is constituted by 2,3-dihydroxytetradecanoic acid. The carbohydrate component is mannose. In hassalidin B, the additional carbohydrate unit, rhammose, is attached to the 3-hydroxyl group of the C_14_-acyl side chain.

Sch 20561 and Sch20562 **21** (Afonso et al. 1999a, b) are major components of a fermentation complex, which shows potent antifungal activity and is produced by the bacterium *Aeromonas* sp. These cyclic nonadepsilipopeptides contain two of the (*E*)-ΔAbu residues. The lipid part of the peptide is constituted by 3-hydroxytetradecanoic acid. Sch20562 has the carbohydrate unit, glucose, attached to the 3-hydroxyl group of the C_14_-acyl side chain. Sch 20561 is the aglycone of Sch20562. The stereochemistry of the ΔAbu residues was deduced on the basis of a comparison of ^1^H NMR chemical shifts of the studied peptides and small synthesised model compounds, isomers *Z* and *E* of *N*-acetyldehydrobutyrine methyl ester (Ac-ΔAbu-OMe). The structural similarity of the peptides **19-21** should be noted. These are cyclic nonapeptides, with five identical amino acids, the lipid part constituted by 3-hydroxytetradecanoic acid, and glycosylated via the hydroxyl group of threonine or fatty acid. Therefore, they can be perceived as a family of compounds. These are also examples of occurrences in nature of the isomer *E* of the ΔAbu residue.

Acculitins A–C **22** (Bewley et al. 1996), isolated from the lithistid sponge *Aciculites orientalis*, inhibit the growth of *Candida albicans* and are cytotoxic towards the HCT-116 (colon carcinoma human cell line). They are bicyclic glycopeptidolipids, containing ten amino acid residues, including two (*E*)-ΔAbu as determined by ROESY. The carbohydrate moiety, d-lyxose, is attached at the 3-positon of C_13_–C_15_ 2,3-dihydroxy-4,6-dienoic acid. The acculitins A–C differ in length of this fatty acid. From the same source two other closely related compounds, aciculitamides A and B **23,** were isolated (Bewley et al. 1996). The structural differences are in imidazole ring and geometry of one ΔAbu residue: *E* and *Z* for aciculitamides A and B, respectively.

Bogorols **24** (Barsby et al. 2001, 2006) have been isolated from cultures of a marine *Bacillus* sp. This family of peptide antibiotics is active against vancomycin-resistant *Enterococcus* spp. as well as methicilin-resistant *Staphylococcus aureus*, common Gram-positive human pathogens. Bogorols are linear dodecapeptides containing a number of structural features, which include a reduction of the C-terminal residue to valinol, a presence of N-terminal 2-hydroxy-3-methylpentanoic acids, the incorporation of four d-amino acids, and a presence of the (*E*)-ΔAbu residue. The configuration *E* was determined by NOESY. The structural differences between bogorols rely on the changes in sequence at positions 2 and 4. Three potentially charged residues make bogorols cationic peptide antibiotics.

Largamide H **25** (Plaza and Bewley 2006) isolated from marine cyanobacterium *Oscillatoria* sp., has a different structure from largamides A–C **16**. This cyclodecadehydropeptide contains two ΔAbu residues, both in configuration *Z* and *E*, as deduced from the ROESY spectrum. Largamide H was tested for toxicity towards HCT-116 cell line, but no inhibitory activity was observed.

Pahayokolides A and B **26** are cycloundecapeptides isolated from cyanobacterium *Lyngdybya* sp. (An et al. 2007). They possess two ΔAbu resides with both *Z* and *E* configurations as determined by NOE. The structural difference between pahayokolides A and B is *N*-acetyl-*N*-methyl-leucine at the 3-amino-2,5,7,8-tetrahydroxy-10-methylundecanoic acid moiety. Tests for pahayokolide A revealed a broad spectrum of cytotoxicity, including an inhibition of a number of cancer cell lines (Berry et al. 2004).

Laxaphycin A **27** (Frankmolle et al. 1992a, b; Bonnard et al. 1997, 2007), isolated from fresh water cyanobacterium *Anabea laxa* and marine cyanobacterium *Anabea torulosa*¸ lobocyclamide A (MacMillan et al. 2002) from cyanobacteria *Lyngbya confervoides*, and hormothamnin A (Gerwick et al. 1992, 1989) from marine cyanobacterium *hormothamnion enteromorphoides* are a family of cycloundecapeptides. Lobocyclamide A is the [l-Ser^2^, d-Tyr6, l-*allo*-Ile^9^] laxaphycin A analogue. Both compound possess the (*E*)-ΔAbu residue as determined by NOE. Hormothamnin A differs from laxaphycin A only in the geometrical isomer (*Z*) of the ΔAbu residue. These peptides reveal cell-growth inhibitory (antifungal, anticancer) activity. Laxaphycin A is rather low toxic at inactive concentrations; it strongly potentiates the cell toxicity of laxaphycin B, a closely related structural analogue. This indicates a synergistic effect whose efficacy increases with increasing concentration of laxaphycin A. Similarly, lobocyclamides A and B displayed synergistic in vitro antifungal activity. It should be noticed that contrary to laxaphycin A, hormothamnin A is highly toxic on a variety of solid cancer cell lines (Gerwick et al. 1989). As the only difference between laxaphycin A and hormothamnin A is configuration of the ΔAbu residue (*E* and *Z*, respectively), it is likely that the configuration of dehydrobutyrine plays a critical role in the higher cytoxicity of hormothamnin A (Bonnard et al. 2007).

Tumescenamides **28** were isolated from the fermentation broth of a marine bacterium, *Streptomyces* (Motohashi et al. 2010; Kishimoto et al. 2012). These are cyclopentalipodepsipeptides containing the (*Z*)-ΔAbu residue with C-terminal ester bond. Stereochemistry was obtained from 2D NMR data. Tumescenamides A and B differ in the type of fatty acid. Tumescenamides A and C differ only in the configuration of the valine residue. Tested so far, tumescenamide A showed weak activity only in the luciferase-reporter assay system, which enabled observing the expression of insulin-degrading enzyme (IDE). Tumescenamide C exhibited antimicrobial activity with high selectivity against the *Streptomyces* species.

An interesting example is phomalide **29**, a selective phytotoxin produced by the fungus *Leptosphaeria maculans*, responsible for leaf spot and stem cankers (blackleg), one of the most damaging diseases of oilseed *Brassicas*, particularly canola (Howlett et al. 2001). Phomalide is a cyclic pentadepsipeptide containing the (*E*)-ΔAbu residue with *C*-terminal ester linkage (Pedras and Taylor 1993). Biological evaluations of phomalide and its synthetic analogues, isophomalide having (*Z*)-ΔAbu residue and dihydrophomalides, revealed that only phomalide caused necrotic, chlorotic, and reddish lesions on canola (*Brassica napus* and *Brassica rapa*; susceptible to blackleg) (Ward et al. 1999). This is another example, which shows that not only the presence of the dehydroamino acid residue, but also that its proper geometric isomer is crucial for the biological activity of the peptide.

### Dehydrovaline and dehydroisoleucine

Lasiodine A **30** (Marchand et al. 1969) is a linear tetrapeptide isolated from the leaves of *Lasiodiscus marmoratus*, a small plant genus in the family *Ramnaceae*. The biological activity of lasiodine A remains unknown. This was the first report on dehydrovaline residue (ΔVal) in a natural compound. Additionally, it is also a rare example of plant as a source of dehydropeptide. Both the source of origin as well as alkylated amino groups provide a basis to classify lasiodine A as an alkaloid peptide.

FR225659 and FR225656 **31** (Zenkoh et al. 2003) are linear *N*-acyl tripeptides produced by the fungi *Helicomyces* sp. All residues are non-standard: 3-chloro-4-hydroxyarginine, 3-hydroxy-3-methylproline, and ΔVal or dehydroisoleucine ((*E*)-ΔIle), respectively, for FR225659 and FR225656. The isomer *E* of ΔIle was shown by NOE. Both compounds are inhibitors of gluconeogenesis and thus may be useful as anti-diabetic agents (Ohtsu et al. 2003a, b).

Yaku’amides A and B **32** (Ueoka et al. 2010) from the marine sponge *Ceratopsion* sp. are cytotoxic linear peptides. These tridecapeptides contain four α,β-dehydroamino acid residues: (*Z*)-ΔIle1, (*E*)-ΔIle2, (*Z*)-ΔIle3, and ΔVal. The geometries of the double bonds in the three ΔIle residues were determined on the basis of NOESY data as well as total synthesis (Kuranaga et al. 2013). Yaku’amide B has Ala in place of the Gly residue. Yaku’amides exhibit potent cell-growth inhibitory activity against P388 murine leukaemia cells.

Antrimycin **33** (Morimoto et al. 1981) was isolated from bacteria *Streptomyces xanthocidicus*. It shows antibiotic activity against *Mycobacterium smegmatis* (Shimada et al. 1981). This linear heptapeptide contains four unusual amino acid residues including (*E*)-ΔIle. To confirm the structure, the fragment of peptide was hydrogenolysed under atmospheric pressure of hydrogen with platinum as catalyst and then hydrolysed. The presence of isoleucine and the only trace of *allo*-isoleucine proved the geometrical isomer *E*. Year after cirratiomycins A and B (Shiroza et al. 1982a, b, c) were isolated from bacteria *Streptomyces cirratus*. These antibiotics exhibit activities against *Lactobacillus case* as well as some strain of *Mycobacterium* and *Streptococcus*. The analysis of the structures shows that cirratiomycin A is [Leu^5^] antrimycin and cirratiomycin B is homologous with antrimycin.

### Dehydroproline

Several species of *Streptomyces* produce a broad family of virginiamycin antibiotics. These compounds are individually of rather low activity, but they show a strong synergism against Gram-positive bacteria (Cocito 1979; Dang et al. 2004). Ostreogrycin A (virginamycin M1) **34** (Delpierre et al. 1966; Bycroft 1977; Durant et al. 1974) is a small peptide with a 23-membered lactone ring and α,β-dehydroproline (ΔPro) with C-terminal ester bond. The literature relating to this compound is complicated by the plethora of synonyms which exist for the individual members of each family (mikamycin A, PA114A, pristinamycin II A, streptogramin A, vernamycin A) (Kingston et al. Kingston and Kolpak 1980).

### Dehydroaspartic acid

Phomopsin A **35**, mycotoxin produced by the fungus *Phomopsis leptostromiformis*, is responsible for lupinosis disease in animals (Culvenor et al. 1983). X-ray analysis shows that it is a linear hexapeptide containing a 13-membered ring with an ether bridge (Mackay et al. 1986). The unique structure of phomopsin A does not contain any standard amino acid. The presence of four dehydroamino acid residues, (*E*)-dehydroaspartic acid ((*E*)-ΔAsp), (*E*)-ΔIle, as well β,γ-dehydro-l-valine and β,γ-dehydro-l-proline, makes phomopsin A one of the most dehydrogenated of dehydropeptides. To date, this is the only example of occurrence of dehydroaspartic acid in nature.

### Dehydrotryptophan

Telomycin **36** (Sheehan et al. 1968) is a bacterial *Streptomyces* metabolite active against Gram-positive organisms. The structure contains 11 amino acids: nonapeptide lactone and short dipeptide chain. The dehydrotryptophan residue (ΔTrp) is suggested for the chromophore of telomycin. The stereochemistry, including geometrical isomer of ΔTrp, has not been determined. Closely related compounds A-128-OP and A-128-P **37** were obtained from natural antibiotic neotelomycin (Belova and Stolpnik 1966). The amino acid composition of A-128-OP is very similar to that of telomycin. Threonine is incorporated in the side chain instead of the lactone ring. Aspartic acid creates an α-carboxypeptide bond. In A-128-P, the l-*trans*-3-hydroxyproline is replaced by l-proline. The configurations of the amino acids were determined by the highly active d-amine oxidase (Silaev et al. 1971). The stereochemistry of ΔTrp was not determined. Nevertheless, it was shown that the modification of tryptophan residues led to a considerable decrease or even loss of bioactivity (Katrukha et al. 1974). The marine derived halotolerant fungal strain PT06-1, identified as *Aspergillus sclerotiorum*, was isolated from the Putian Sea, Salt Field, Fujian, China. Sclerotides A and B **38** (Zheng et al. 2009) were identified from the metabolites and show moderate antifungal activity. These cyclohexapeptides contain the ΔTrp residue with geometry *Z* for sclerotide A and *E* for sclerotide B. Stereochemistry of the ΔTrp residue was determined using the 2D NMR technique, a comparison of chemical shifts to the ΔTrp containing diketopiperazine isoechinulin (Wang et al. 2007) as well as theoretical calculations. Both sclerotides, A and B, are stable in dark and photointerconvertible into each other in light. The isomer Z is more stable. The equilibrium of sclerotides A and B is 87:13 with little influence of both temperature and solvent.

The Okinawan marine sponge *Theonella* sp. is the source of many unique peptides, amongst others keramamides. Keramamide F **39** (Itagaki et al. 1992), a cyclic heptapeptide, contains the (*Z*)-ΔTrp residue with the geometry determined by HMBC and NOESY. Keramamide F is an anticancer compound as it shows cytotoxicity against human epidermoid carcinoma KB cells and murine lymphoma L1210 cells.

Janthinocins A–C **40** are antibacterial agents produced by *Janthinobacterium* *lividium*. They are active against aerobic and anaerobic Gram-positive bacteria and are more potent than vancomycin (O’Sullivan et al. 1990; Johnson et al. 1990). Janthinocins are cyclodecadepsipeptides containing ΔTrp as well as ΔAbu residues. The structural differences between janthinocins A–C occurs in position 8, where there is *threo*-β-hyroxytryptophan, β-ketotryptophan, and dehydrotryptophan (ΔTrp) residues, respectively. The stereochemistry of dehydroamino acid residue was not determined.

### Unique dehydroamino acids

This part describes the naturally occurring peptides, which contain the α,β-dehydroamino acid residues having unique side chains.

Cyrmenins **41** (Sasse et al. 2003; Leibold et al. 2004) were isolated from the culture broth of strains of the myxobacteria *Cystobacter armeniaca* and *Archangium gephyra* (myxobacteria). Cyrmenins reveal antifungal activity and thus can be potentially important for the agricultural industry. These linear dipeptides consist of N-terminal unsaturated fatty acids and two adjacent dehydroamino acids, ΔAla and (*Z*)-O-methyl-dehydroserine (ΔSer(Me)). This makes them a unique series of natural products. A short and efficient synthesis of cyrmenin B1 has been published (Chakor et al. 2009). The modifications of the cyrmenins by introduction of alanine or serine in place of dehydroalanine as well as the replacement of the methoxy group with a hydrogen atom resulted in a complete loss of antifungal activity (Chakor et al. 2012).

Victorins **42** are a family of toxins produced by the fungus *Cochliobolus victoriae*, which causes victoria blight of oats (Wolpert et al. 1985, 1986; Durow et al. 2009). Victorin C is the main component of this toxin complex. These pentapeptides consist of a short dipeptide chain and a 12-membered ring containing a unique (*E*)-β-chlorodehydroalanine residue ((*E*)-Δ(βCl)Ala). The geometry of this residue was established using a combination of synthesis and NMR methods (Durow et al. 2009).

Tuberactinomycins **43** are a family of tuberculostatic antibiotics which are produced by bacteria *Streptomyces* (Yoshioka et al. 1971; Noda et al. 1972). These cyclic hexapeptides contain unusual amino acid residues, such as α,β-diamino propionic acid and β-ureidodehydroalanine (Δ(βU)Ala). The geometry of Δ(βU)Ala is *Z*. The structural differences rely on a combination of two pairs of amino acid residues: l-tuberactidine/l-capreomycidine and l-β-lysine/γ-hydroxy-l-β-lysine. Tuberactinomycin B is a homologue of viomycin, the structure of which was determined by X-ray techniques (Bycroft 1972). Similar in structure and biological activity are capreomycins **44** (Nomoto et al. 1977). The branched part constituted by the β-lysine residue is linked with the β-amino group of α,β-diaminopropionic acid residue in a different position than in tuberactinomycins.

Sponge *Callyspongia abnormis* and *Callyspongia aerizusa* are a source of callynormine A (Berer et al. 2004) (with the structure elucidated by interpretation of NMR data and X-ray diffraction analysis), like also callyaerins A–H **45** (Ibrahim et al. 2008, 2010). These compounds are cyclic peptides containing from 9 to 14 amino acid residues. The core of their structure is unusual (Z)-2,3-diaminoacrylic acid, which provides the template for ring closure (5-8 residues) and supports the linkage to the peptide side chain (3-5 residues), which is always initiated by a proline moiety. The amino acid residues are predominantly hydrophobic and all in the L form. The bioactivity of callynormine A is not known, but callyaerins reveal various cytotoxicities: antibacterial, antifungal, and antitumour.

Another sponge *Cliona celata* is the source of celenamides **46** (Stonard and Andersen 1980a, b). Celenamides A–C are linear tripeptides containing leucine or valine, 3,4,5-trixydroxydehydrophenylalanine, and 6-bromotryptophan. Celenamide D has leucine and two 3,4,5-trixydroxyphenylalanines. The geometry of this dehydroamino acid was not determined. The biological activity also remains unknown. However, celenamide E, a closely related dipeptide and a possible biosynthetic precursor of the previously reported celenamides A–C, was isolated from the Patagonian sponge *Cliona chilensis* (Palermo et al. 1998). The stereochemistry of the 3,4,5-trixydroxyphenylalanine was established as *Z* by NMR techniques. Celenamide E shows antibiotic activity against Gram-positive bacteria. Thus, it can be supposed that these are the features of other compounds in this family. Because of free amino group at the N-terminus, celenamides are classified to linear peptide alkaloids.

Tunichromes **47** are dipeptides containing one or more dehydrodopa-derived units that have been identified in the blood cells of at least 11 species of tunicates (Cai et al. 2008). Tunichromes An-1, An-2, An-3, and Mm-1, Mm-2 have been extracted and characterised from blood cell lysates of the phlebobranch *Ascidia nigra* and the stolidobranch *Molgula manhattensis* (Bruening et al. 1985; Oltz et al. 1988). Tunichromes An-1, An-2, An-3 contain 3,4,5-trixydroxydehydrophenylalanine, whereas tunichromes Mm-1 and Mm-2 contain 3,4-dixydroxydehydrophenylalanine. The geometry of these dehydroamino acids was not determined. Tunichromes exhibited antimicrobial activity against Gram-negative bacteria *Escherichia coli* and *Photobacterium phosphorium*. The oxidation products of tunichromes possess inherent cross-linking properties. Hence, it is possible that tunichromes participate in tunic production by forming adducts and cross-links with structural proteins and/or carbohydrate polymers (Cai et al. 2008). It is also suggested that tunichromes, specific blood pigments of marine tunicates, could play a role in sequestering and reducing vanadium or iron (Oltz et al. 1988).

Azinomycins A and B **48** are antitumour antibiotics produced by culture broth *Streptomyces* species (Yokoi et al. 1986; Ishizeki et al. 1987). These compounds, structurally and mechanistically unrelated to other families of antitumour agents, contain only one amino acid residue: unusual 1-azabicyclo[3.1.0]hexane (aziridino[1,2-*a*]pyrrolidine) ring system appended as part of a dehydroamino acid. The stereochemistry of this dehydroamino acid was studied by NOE experiment including dihydro derivative obtained by catalytic hydrogenation (Yokoi et al. 1986). This unique ring system has been reported to be the reason for the DNA cross-linking abilities and cytotoxicity of these metabolites. The azinomycins have also shown bioactivity against a range of Gram-negative and Gram-positive bacteria (Foulke-Abel et al. 2011).

Dityromycin **49** was isolated from the culture broth of the soil microorganism *Streptomyces* (Ōmura et al. 1977). This bicyclic decadepsipeptide contains two unique α,β-dehydroamino acid residues: 2-amino-3-hydroxymethyl-4,5-epoxy-α,β-dehydropentanoic acid as well as *O*-aryl-*N*-methyldehydrotyrosine with C-terminal ester linkage (Teshima et al. 1988). The configurations of these moieties have not been clarified yet. A new antibiotic GE82832, a translocation inhibitor, has recently been reported (Brandi et al. 2012). Although its structure had not been completely solved, similarities in the MS spectra like also in the inhibitory activities indicate that GE82832 is highly related to dityromycin.

### Main chain modifications

The structural changes within the α,β-dehydroamino acid residue concern not only the side chain, but also the main chain.

### *N*-Methyldehydroamino acids

The cyanobacteria (blue-green algae) produce a hepatotoxic family of cyclic heptapeptides originally described as cyanoginosins (Botes et al. 1984, 1985), but presently called microcystins **50** according to *Microcystis*, the first genera of cyanobacteria associated with their biosynthesis (Pearson et al. 2010; Merel et al. 2013; Fujiki and Suganuma 2011). The structure of mycrocystins are characterised by the (*2S*,*3S*,*8S*,*9S*)-3-amino-9-methoxy-2,6,8-trimethyl-10-phenyldeca-4,6-dienoic acid (Adda) as well as *N*-methyldehydroalanine residue, Δ(Me)Ala. There are over 90 compounds classified as microcystins (Łukomska et al. 2002; Welker and von Dohren 2006). The variability occurs most often at the positions 2 and 4 (Carmichael et al. 1988), although the numbering does not correspond to the suite of biosynthetic steps (Tillett et al. 2000). The Δ(Me)Ala residue plays an important role in the toxicity of mycrocystins. The carbon–carbon double bond reacts with the thiol function of protein phosphatase 1 through Michael addition, binding covalently mycrocystin to this enzyme (Goldberg et al. 1995). There are also microcystins, which contain non-methylated ΔAla (Namikoshi et al. 1992a, b; Luukkainen et al. 1994; Sivonen et al. 1992a, b) and both isomers of ΔAbu (Sano and Kaya 1998; Sano et al. 1998; Blom et al. 2001; Beattie et al. 1998) instead of Δ(Me)Ala. Microcystin-LR has an extremely lethal dose response LD50 = 32.5 μg/kg in mice (Schaeffer et al. 1989). However, for non-methylated [ΔAla^7^]microcystin-LR, LD50 equals 250 μg/kg (Namikoshi et al. 1992a, b), whereas for dihydromicrocystin-LR, LD50 equals 85–100 μg/kg (Namikoshi et al. 1993). It can be concluded, therefore, that both the unsaturated functionality and methylation can influence bioactivity. Moreover, it was shown that [d-Asp^3^,(*E*)-ΔAbu^7^]microcystin-RR reveals a higher specific toxicity when compared with the Δ(Me)Ala residue containing microcystins (Blom et al. 2001). This indicates that the kind of dehydroamino acid residue can also be important.

The Δ(Me)Ala residue can be also found in cyclodepsipeptide FR900359 **51**, as confirmed by X-ray structure (Miyamae et al. 1989). This peptide, isolated from the evergreen plant *Ardisia crenata sims* (Myrsinaceae), shows the inhibition of platelet aggregation and a decrease in blood pressure (Fujioka et al. 1988). Closely related compounds denoted as YM-254890, YM-254891, YM-254892, and YM-280193 (Taniguchi et al. 2003a, b, 2004) were isolated from a culture strain of the broth *Chromobacterium* sp. QS3666 (Taniguchi et al. 2003a, b). YM-254890 is a specific Gαq/11 inhibitor on thrombosis and neointima formation (Kawasaki et al. 2005). Semi-synthetic saturated analogues show that the presence of Δ(Me)Ala is not critical for activity (Taniguchi et al. 2003a, b). FR900359 differs from YM-254891 only in one amino acid residue (alanine instead of valine) as well as the acyl group in the short chain. It is interesting that such similar compounds originate from quite different biological sources.

BE-22179 **52** was isolated from the culture broth of *Streptomyces* sp. (Okada et al. 1994; Yoshinari et al. 1994). It constitutes the newest member of the class of naturally occurring, twofold symmetric bicyclic octadepsipeptides (Dawson et al. 2007). The characteristic feature of BE-22179 in comparison to other compounds in this class is the presence of the unusual junction of the Δ(Me)Ala residue with thioester linkage (Boger and Ichikawa 2000). BE-22179 exhibits activity against Gram-positive bacteria (including *S. aureus*), but is inactive against Gram-negative bacteria (Okada et al. 1994). It is also a highly potent inhibitor of DNA topoisomerase II. It was shown to bind to DNA by a high-affinity bisintercalation (Boger et al. 2001). It should be noted that the previously described dityromycin **49** (Teshima et al. 1988) contains *O*-arylated *N*-methyl-dehydrotyrosine with C-terminal ester bond. Both examples, BE-22179 and dityromycin, show that within single amino acid residue at least three structural modifications can be found: α,β-double bond, *N*-methylamide group, and C-terminal ester group.

Nodularins **53** are cyclic pentapeptides produced by cyanobacteria *Nodularia* (Rinehart et al. 1988; Sandstrom et al. 1990; Namikoshi et al. 1994; De Silva et al. 1992; Beattie et al. 2000; Saito et al. 2001). Nodularins reveal similar hepatoxicity as microcystins as well as certain structural similarity, i.e. the presence of Adda and dehydroamino acid residue, which in this case is (*Z*)-*N*-methyldehydrobutyrine residue, (*Z*)-Δ(Me)Abu. Motuporin, although isolated form the crude extract of the marine sponge *Theonella swinhoei* Gray (De Silva et al. 1992), also belongs to this family. Dehydroamino acid is important, but not crucial for biological activity. Reduction of (*Z*)-Δ(Me)Abu residue to dehydronodularin results in a decrease of toxicity (LD50 = 60 and 150 μg/kg, respectively) (Namikoshi et al. 1993).

Tentoxin **54** is a phytotoxic metabolite of the pathogenic fungus *Alternaria tenuis* (Fulton et al. 1965; Saad et al. 1970). It binds to the chloroplast F_1_-ATPase, as it was shown by the crystal structure of the tentoxin-inhbited CF_1_-complex (Groth 2002). Tentoxin is cyclotetrapeptide with *N*-methyl-(*Z*)-dehydrophenylalanine residue, (*Z*)-Δ(Me)Phe (Meyer et al. 1975). It should be noted that acyclic analogues of tentoxin have low, but significant chlorosis activity, whereas both (Z)-ΔPhe residue and N-methyl group are necessary for full activity (Edwards et al. 1996). Moreover, isotentoxin, the semi-synthetic isomer *E* of tentoxin, seems to have no chlorosis activity (Liebermann et al. 1996). This shows that the junction of structural features: the α,β-double bond, the side chain in proper geometrical configuration, and the methyl group at the N-terminal amide bond, creates unique properties necessary for the biological action of this peptide.

The Papua New Guinea sponges *Theonella* produce cycloheptapeptides mutremdamide A **55** (Plaza et al. 2010). Mutremdamide A failed tests to inhibit HIV Entry as well as the growth of *Candida albicans*. Thus, its biological function remains unknown. However, it is the first example of the natural peptide where the α,β-dehydroamino acid residue ((Z)-ΔAbu) is methylated at the C-terminal amide bond.

### Thiazole-, thiazoline-, and oxazole-dehydroamino acids

Thiopeptides are a broad family of sulphur-containing macrocyclic peptides, produced by Gram-positive bacteria, mostly *Streptomyces*. Thiopeptides display activity against Gram-positive bacteria; thus, they attract constant interest as potential antibiotics. The classification, isolation, structural elucidation, biological properties, biological origin, and methods of synthesis have already been extensively reviewed (Bagley et al. 2005; Just-Baringo et al. 2014). The central point of the structure of thiopeptides is pyridine, piperidine, dehydropiperidine, or dehydroimidazopiperidine, which constitute a macrocyclic ring and, in most cases, is substituted by a short linear chain. The macrocyclic part of thiopeptides contains from one to six thiazole rings, like also other heterocyclic rings such as thiazoline, oxazole, 5-methyloxazole, indole, and pyrrolidine. Thiopeptides are also rich in dehydroamino acid residues, which makes them one of the most highly modified peptides.

The presence of heteroaromatic moieties and dehydroamino acid residues makes a specific structural junction, always at the C-terminus of the dehydroamino acid residue. This is caused, most probably, by the requirement of the cyclisation reaction, in which the thiol or hydroxyl group of cysteine, serine, or threonine react with the preceding amide group (Dunbar and Mitchell 2013). Cyclothiazomycins **56** (Aoki et al. 1991; Hashimoto et al. 2006; Mizuhara et al. 2011) contains thiazole-dehydroalanine. Thiazole-(Z)-dehydrobutyrine constitutes micrococcins P1-2 **57** (Lefranc and Ciufolini 2009) thiocilins I-III (Shoji et al. 1976, 1981; Aulash and Ciufolini Aulakh and Ciufolini 2011), YM-266183-4 (Nagai et al. 2003; Suzumura et al. 2003) as well as nosiheptide **58** (Pascard et al. 1977). Thiazole-(*E*)-O-methyl-dehydrothreonine was found in closely related nocatiacins (Constantine et al. 2002; Leet et al. 2003; Li et al. 2003), thiazomycins (Jayasuriya et al. 2007; Zhang et al. 2009), MJ347-81F4 A & B (Sasaki et al. 1998), and glycothiohexide α **59** (Northcote et al. 1994). The thiostrepton family of compounds **60** (Dutcher and Vandeputte 1955; Anderson et al. 1970; Bond et al. 2001; Nishimura et al. 1959; Mori et al. 2007; Tori et al. 1981; Miyairi et al. 1970; Hensens and Albers-Schönberg 1983, 1978; Puar et al. 1981) and closely related Sch 40832 **61** (Puar et al. 1998) contain the thiazoline-(Z)-dehydrobutyrine residue, in which the heterocyclic ring is not fully dehydrated. Furthermore, oxazole- and 5-methyl-oxazole-dehydroamino acids such as oxazole-dehydroalanine, 5-methyl-oxazole-dehydroalanine, oxazole-dehydrobutyrine, 5-methyl-oxazole-dehydrobutyrine, oxazole-dehydrohomobutyrine, 5-methyl-oxazole-dehydrohomobutyrine, oxazole-dehydroleucine, and 5-methyl-oxazole-dehydrohomoserine were found in geninthiocin **62** (Yun et al. 1994a, b, c), berninamycins (Liesch and Rinehart 1977; Abe et al. 1988; Kodani and Ninomiya 2013), sulfomycins **63** (Egawa et al. 1969; Kohno et al. 1996; Vijaya Kumar et al. 1999), promoinducin (Yun and Seto 1995), thiotipin (Yun et al. 1994a, b, c), A10255 **64** (Boeck et al. 1992; Debono et al. 1992; Favret et al. 1992), tioaxamycin (Yun et al. 1994a, b, c), radamycin (Castro Rodríguez et al. 2002), and TP-1161 (Engelhardt et al. 2010). Oxazole-dehydroalanine and oxazole-dehydrobutyrine were also found, respectively, in mechercharmycin A **65** (IB-01211) (Kanoh et al. 2005; Hernández et al. 2007a, b) [226–228] and urukthapelstatin A (Matsuo et al. 2007), a new class of thiopeptides of antitumour activity, which does not have six-membered heterocyclic moiety.

In most cases, the linear chain of thiopeptides consists of dehydroalanine residues (from 1 upto 4) ending at the C-terminus by amide, ester, or acid group **66**. The dehydroalanine side chain possesses also other thiopeptides such us: GE37468A (Stella et al. 1995), philipimycin (Zhang et al. 2008), thiomuracin A (Morris et al. 2009), baringolin (Just-Baringo et al. 2013), kocurin (Martín et al. 2013), and promothiocins (Yun et al. 2001), which do not have dehydroamino acids in the macrocyclic ring. However, non-modified dehydroalanine residue can be also found in the macrocyclic ring of the presented structures **60–64**. This makes ΔAla one of the most abundant dehydroamino acids.

## Conclusions

**Scheme 2:**
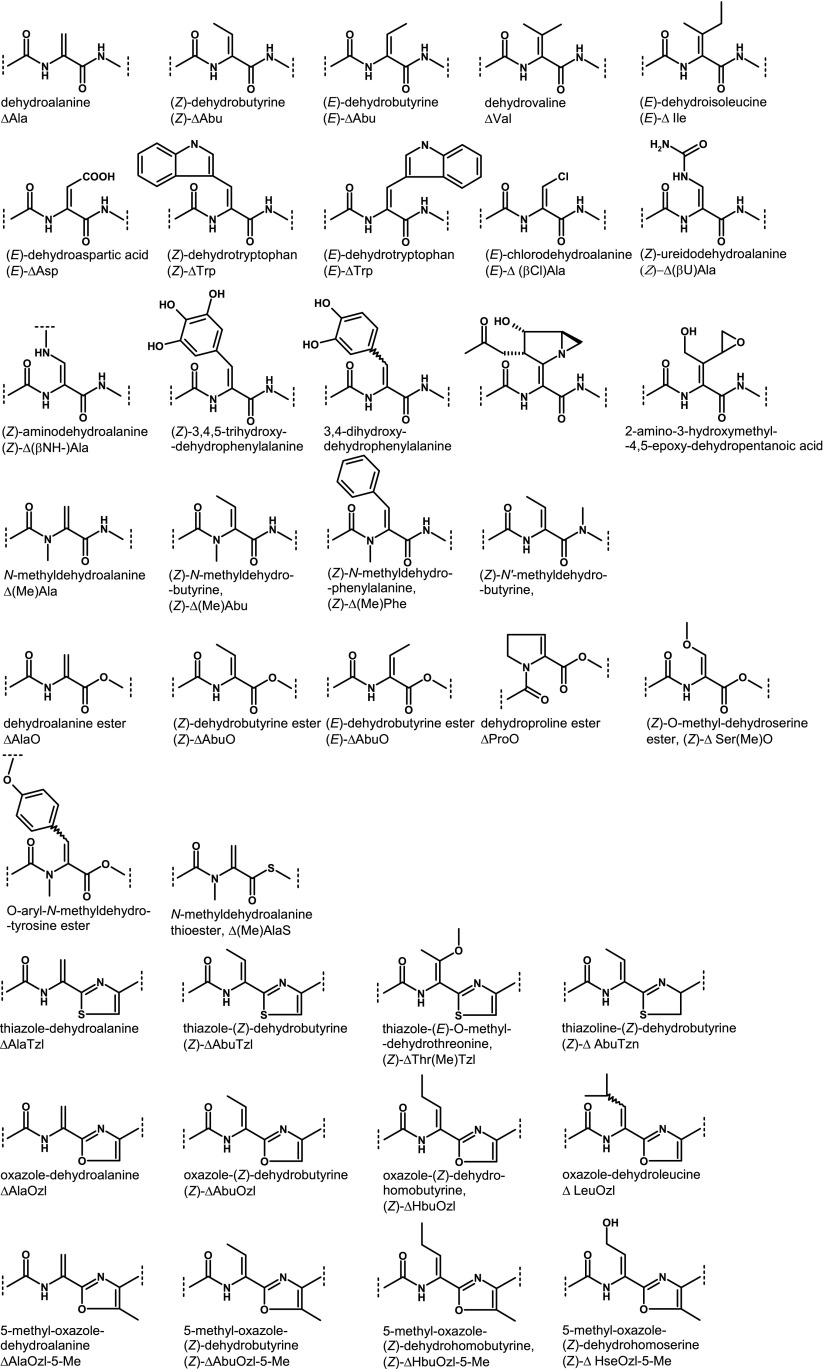
Various structural units of the α,β-dehydroamino acids found in natural peptides; this includes variations of the side chains, geometrical isomers, modifications of main chain, and their combinations

The presented search reveals 37 different structural units of the natural α,β-dehydroamino acids. This includes variations of the side chains, geometrical isomers, modifications of the main chain, as well as combinations of such structural changes (Scheme 2). Amongst dehydroamino acids, dehydroalanine and dehydrobutyrine occur most often, derived from cysteine, serine, and threonine. There are also analogues of other standard amino acids: valine, leucine, isoleucine, proline, aspartic acid, serine, threonine, tryptophan, tyrosine, and phenylalanine. However, there are also α,β-dehydroamino acids with the unique side chain containing chlorine atom, ureido moiety, or epoxy or azirydine rings which cannot be simply derived from the standard amino acids. Interestingly, dehydrophenylalanine, the most often studied α,β-dehydroamino acid, is found only in one natural peptide. Methylation of N-terminal amide bond is one of the main modifications within the dehydroamino acid residue. There is also one example of methylation of the C-terminal amide bond. In nature, the α,β-dehydroamino acid residue can be joined by C-terminal ester or thioester bond. A combination of these modifications occurs. The α,β-dehydroamino acid residue with methylated amide bond at the N-terminus creates the ester or thioester bond at the C-terminus. The α,β-dehydroamino acids are also modified by the presence of C-terminal heterocycle (thiazole, thiazoline, or oxazole)—a common feature of thiopeptides. The presence of the α,β double bond creates a possible *Z*/*E* isomerisation of the side chain. Both isomers are found in nature. Although the thermodynamically more stable isomer Z prevails in case of dehydrobutyrine, it is not so obvious for other residues, which are present clearly in the form of the isomer *E* and for which the stereochemistry has not been determined to date. Few examples indicate that the position of the side chain is crucial for bioactivity, which is an intriguing aspect of the potency of nature. These are excellent examples of structure–activity relationship.

**Scheme 3:**
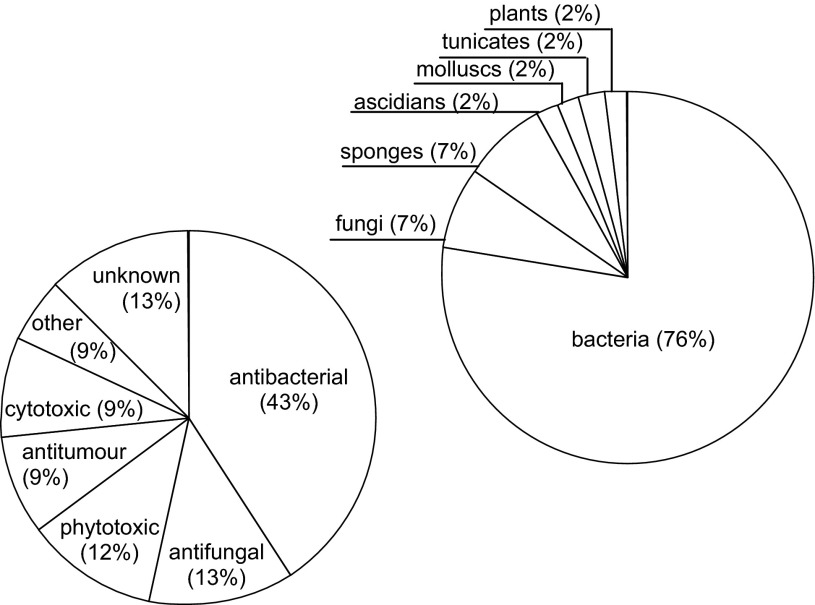
Source of origin and bioactivity of peptides containing the α,β-dehydroamino acids

Peptides containing the α,β-dehydroamino acids are produced primarily by bacteria (Scheme 3). Fungi are the second source. Other organisms such as ascidians, molluscs, sponges, tunicates and even higher plants have been reported. Nevertheless, there is evidence that they are not the origin of the source of dehydropeptides. Peptides containing the α,β-dehydroamino acids reveal various biological activities. Most exhibit antibacterial and antifungal role. Therefore, they are regarded as precursors of new antibiotics. A large group is the phytotoxic pathogens, especially those produced by fungi. Another group reveals promising antitumour activity. Unusual antithrombotic, reducing pigment, or putative metal sequestering features can be also found. Many of the dehydropeptides show a variety of activities. However, the functions of a considerable group of dehydropeptides as well as the α,β-dehydroamino acid residues are still unrecognised.

## Electronic supplementary material

Below is the link to the electronic supplementary material.

Supplementary material 1 (PDF 160 kb)
