# Supplementary material for: α,β-Dehydroamino acids in naturally occurring peptides
Source: Amino Acids. 2014 Oct 17;47(1):1–17. doi: 10.1007/s00726-014-1846-4 (PMC4282715; doi:10.1007/s00726-014-1846-4)

1 nisin

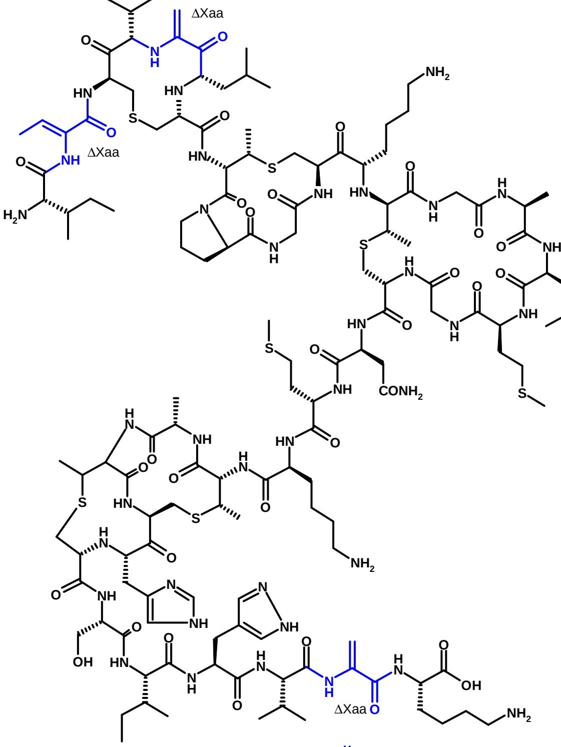

2 AM-toxins

AM-toxin I, R = OCH_3
AM-toxin II, R = H
AM-toxin III, R = OH

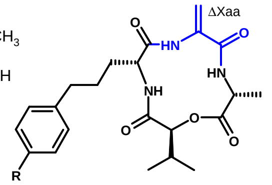

3 vitilevuamide

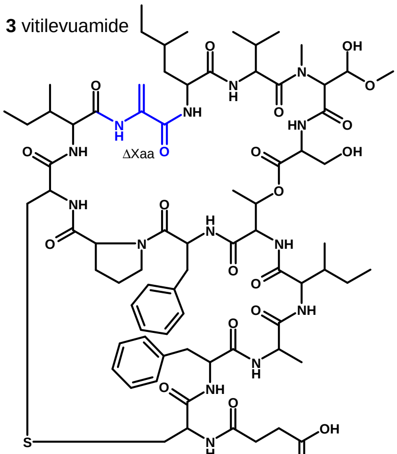

4 corpeptin A

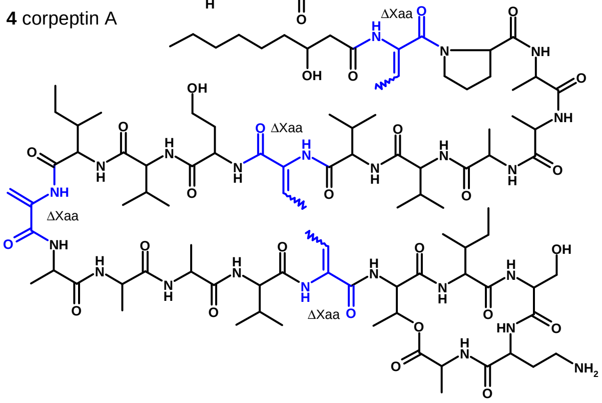

5 tolaasins

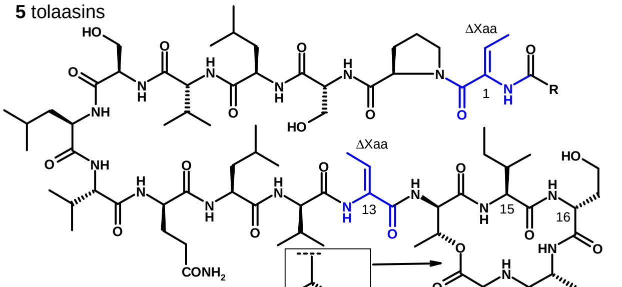

tolaasin I, R = 3-hydroxyoctaonate
tolaasin II = [Gly^{16}]tolaasin I
tolaasin A = tolaasin I,
R = $-(CH_2)_3COOH$
tolaasin B = [Val^{15}]tolaasin I
tolaasin D = [Leu^{15}]tolaasin I
tolaasin E = [Leu^{15}Gly^{16}]tolaasin I

tolaasin C

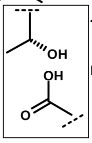

6 fuscopeptins

fuscopeptin A, FA = 3-hydroxyoctanoate
fuscopeptin B, FA = 3-hydroxydecanoate

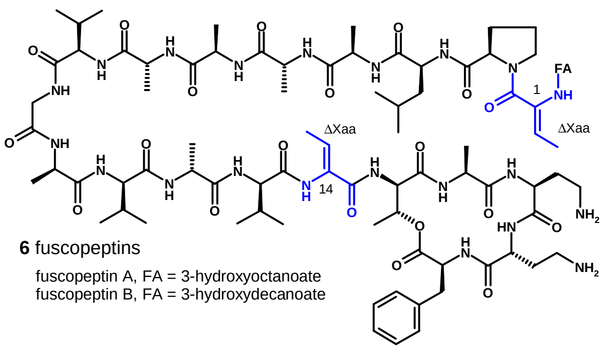

7 syringopeptins 25

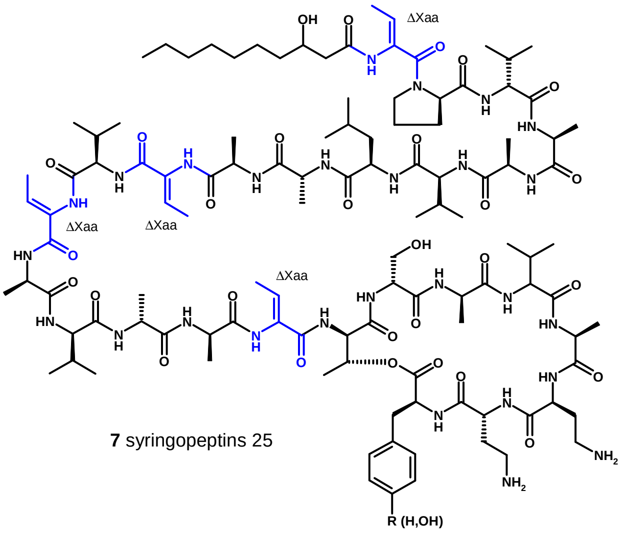

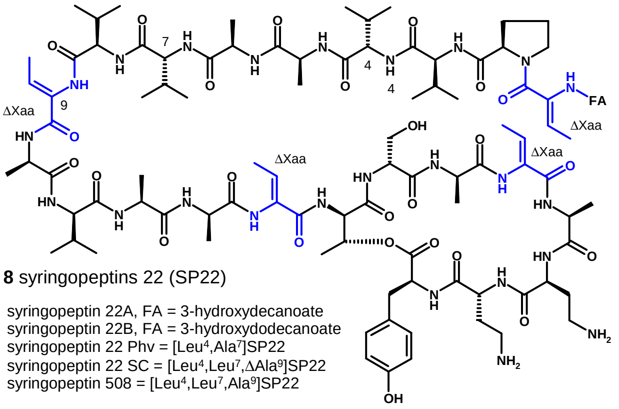

8 syringopeptins 22 (SP22)

syringopeptin 22A, FA = 3-hydroxydecanoate
syringopeptin 22B, FA = 3-hydroxydodecanoate
syringopeptin 22 Phv = [Leu^{4},Ala^{7}]SP22
syringopeptin 22 SC = [Leu^{4},Leu^{7},ΔAla^{9}]SP22
syringopeptin 508 = [Leu^{4},Leu^{7},Ala^{9}]SP22

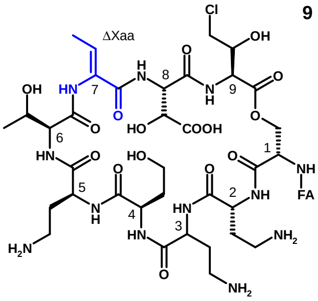

9 syringostatins family

syringostatins
A, FA = tetradecatnoate
B, FA = 3-hydroxytetradecanoate
syringotoxin = [Gly[3]]syringostatin B
syryngomycins = [D-Ser[2],D-Dab[3],L-Dab[4],L-Arg[5],L-Phe[6]]syringostatins
A, FA = 3-hydroxydecanoate
E, FA = 3-hydroxydodecanoate
G, FA = 3-hydroxytetradecanoate
pseudomycins = [L-Asp[3],L-Lys[4],L-Orn[5]]syringostatin
A, FA = 3,4-dihydroxydodecanoate
B, FA = 3-hydroxytetradecanoate
C, FA = 3,4-dihydroxyhexadecanoate
D, FA = 3-hydroxyhexadecanoate
cormycin A = [D-Orn[2],L-Asn[3],L-His[5]]syringostatin

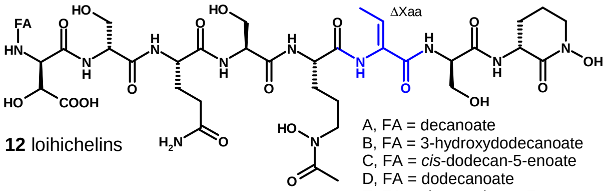

12 loihichelins

A, FA = decanoate
B, FA = 3-hydroxydodecanoate
C, FA = *cis*-dodecan-5-enoate
D, FA = dodecanoate
E, FA = cis-tetradecan-7-enoate
F, FA = tetradecanoate

13 lavendomycin

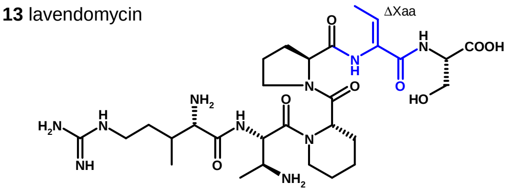

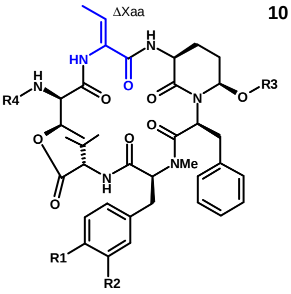

10 dolastatin family

	R1	R2	R3	R4
dolastatin 13	H	H	H	A
symplostatin 2	OH	H	H	B
somamide A	OH	H	H	C
somamide B	OH	H	H	D
lyngbyastatin 4	OH	H	H	E
lyngbyastatin 5	OH	H	Me	F
lyngbyastatin 6	OH	H	H	G
lyngbyastatin 7	OH	H	H	H
lyngbyastatin 8	OH	H	H	I
lyngbyastatin 9	OH	H	H	J
lyngbyastatin 10	OH	Br	H	J
bouillomide A	OH	H	H	K
bouillomide B	OH	Br	H	K
molassamide B	OH	H	H	L

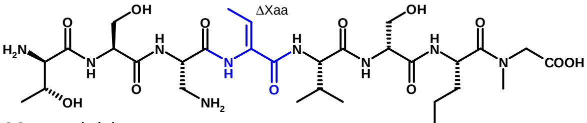

14 stenothricin

15 FK228 (FK901228)

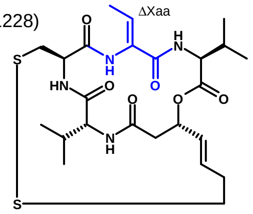

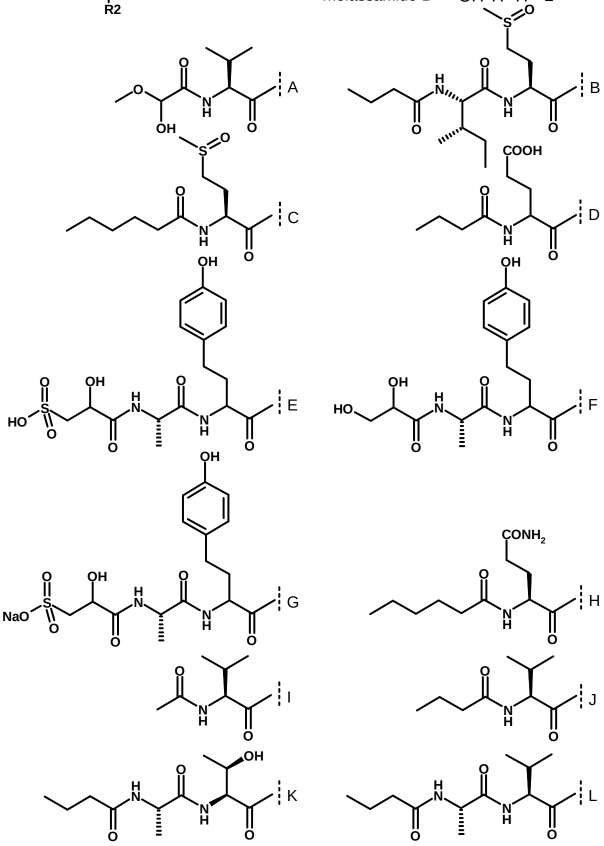

A, B, C, D, E, F, G, H, I, J, K, L

16 largamides

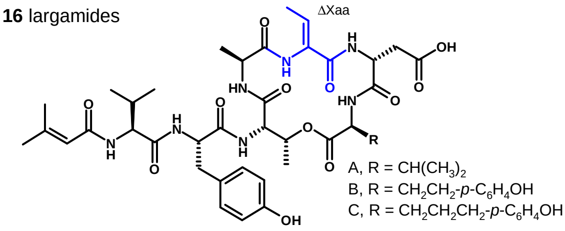

A, R = $CH(CH_3)_2$
B, R = CH_2CH_2-*p*-C_6H_4OH
C, R = $CH_2CH_2CH_2$-*p*-C_6H_4OH

17 kahalalide F

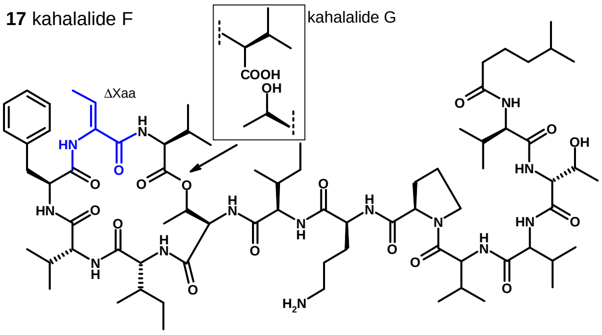

kahalalide G

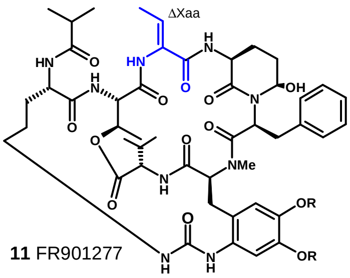

11 FR901277

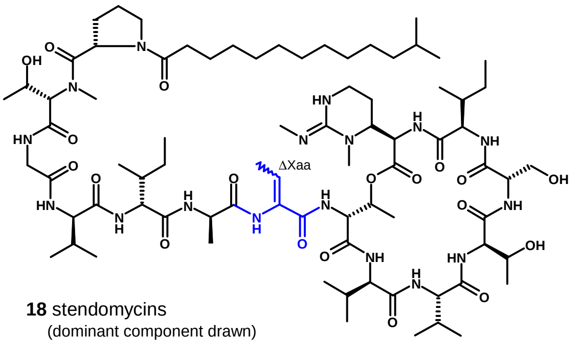

18 stendomycins
(dominant component drawn)

19 herbicolin A

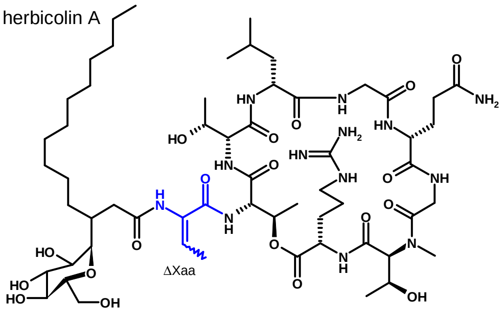

24 bogorols

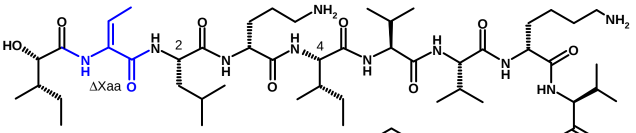

bogorol A (drawn)
bogorol B = [Val^{2}]bogorol A
bogorol C = [Val^{2},Val^{4}]bogorol A
bogorol D = [Met^{2}]bogorol A
bogorol E = [methionine sulfoxie^{2}]bogorol A

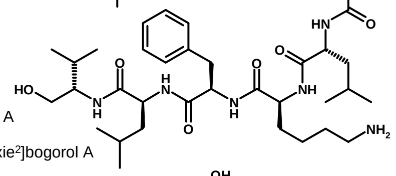

20 hassalidins

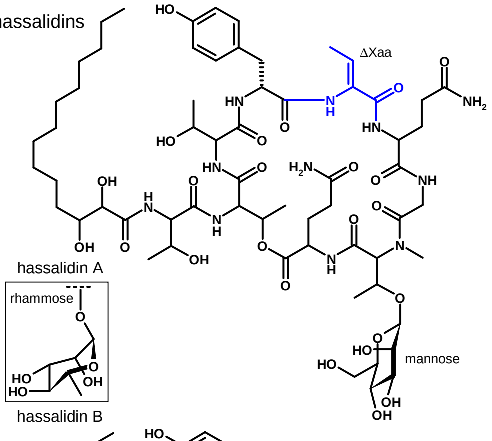

25 largamide H

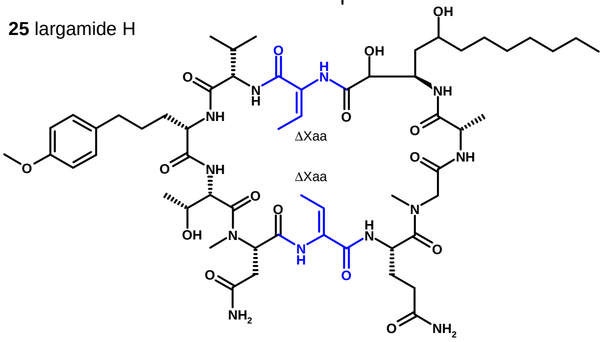

26 pahayokolides

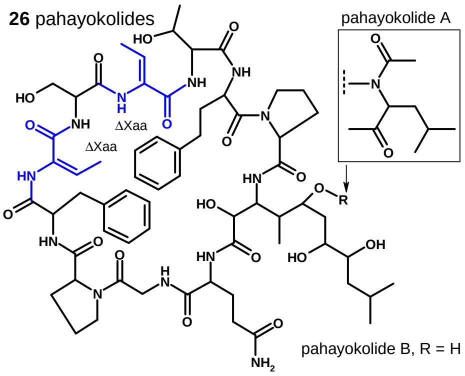

21 Sch 20562

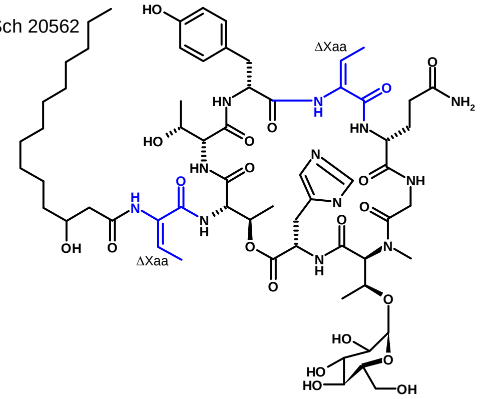

27 laxaphycin A

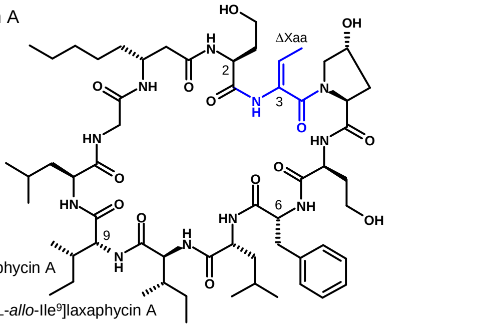

hormothamnin A
([(*Z*)-ΔAbu^{3}]laxaphycin A
lobocyclamide A
([L-Ser^{2}, D-Tyr^{6}, L-*allo*-Ile^{9}]laxaphycin A

22 aciculitins

A, R = C_5H_{11}
B, R = C_6H_{13}
C, R = C_7H_{15}

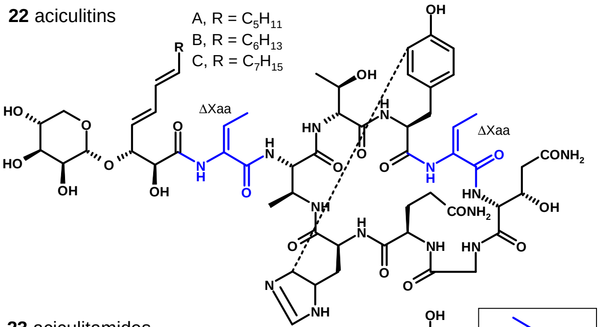

28 tumescenamides

A, n = 2
B, n = 3
C = [L-Val]tumescenamide A

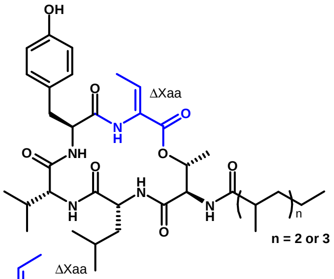

23 aciculitamides

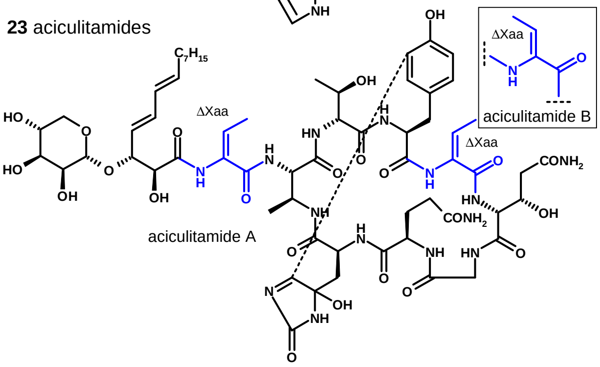

29 phomalide

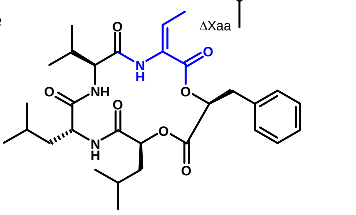

30 lasiodine

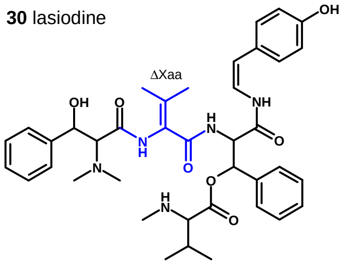

31 FR225659, FR225656

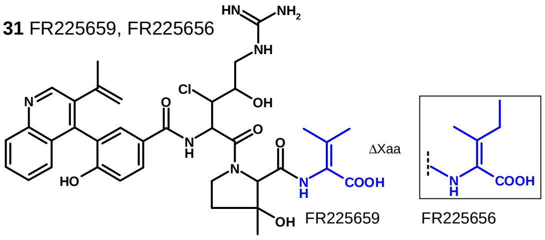

32 yaku'amides

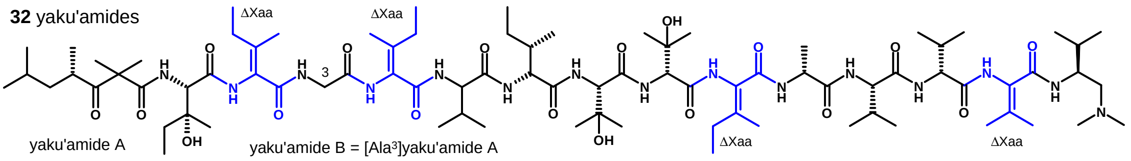

33 antrimycin, cirratiomycins
cirratiomycin A = [Leu5]antrimycin
cirratiomycin B = antrimycin

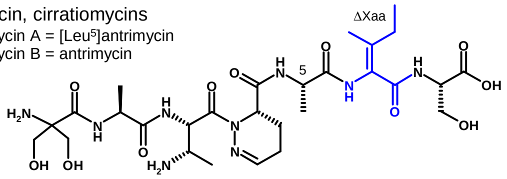

38 sclerotides

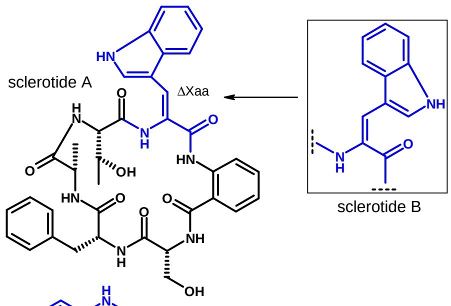

34 ostreogrycin A

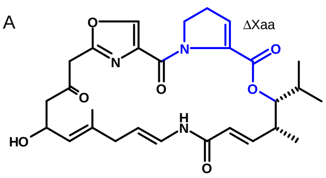

39 keramide F

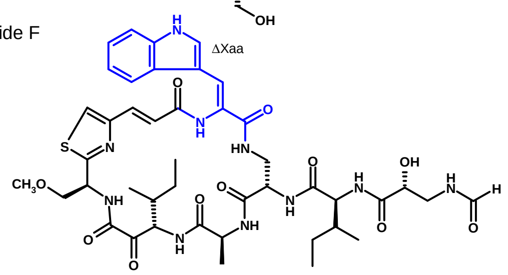

35 phomopsin A

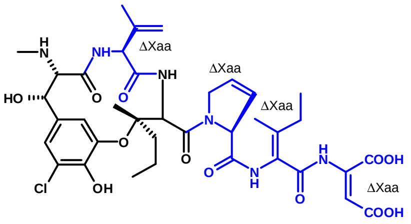

40 janthinocins

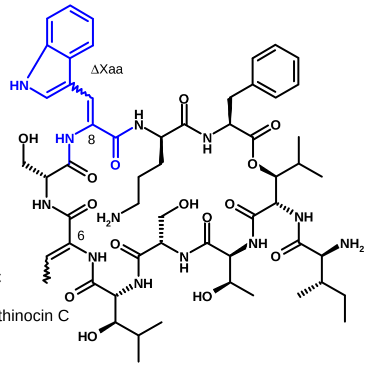

janthinocin C (drawn)
janthinocin B =
[β-ketotryptophan[8]]janthinocin C
janthinocin A =
[*threo*-β-hydroxytryptophan[8]]janthinocin C

36 telomycin

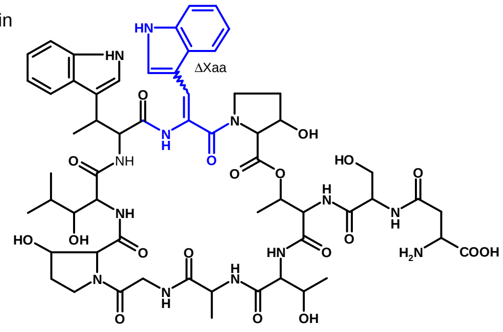

37 A-128-OP, R = OH
A-128-P, R = H

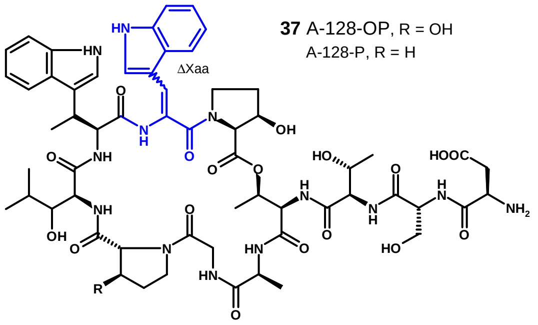

41 cyrmenins

A, R = $CH_2CH(CH_3)_2$
B_1, R = $CH_2CH_2CH_3$
B_2, R = $CH(CH_3)_2$

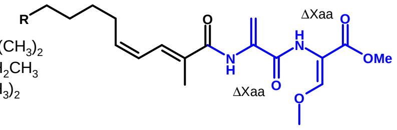

42 victorins

	R1	R2	R3
B,	CH_2Cl,	OH,	$CH(OH)_2$
C,	$CHCl_2$,	OH,	$CH(OH)_2$
D,	$CHCl_2$,	H,	$CH(OH)_2$
E,	CCl_3,	OH,	$CH(OH)_2$
M,	$CHCl_2$,	OH,	CH_2NH_2

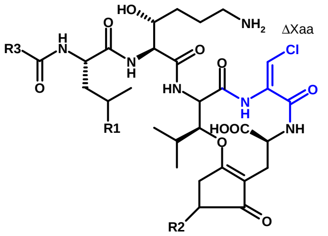

43 tuberactinomycins

A, R1 = OH, R2 = OH
B (viomycin), R1 = H, R2 = OH
N, R1 = OH, R2 = H
O, R1 = H, R2 = H

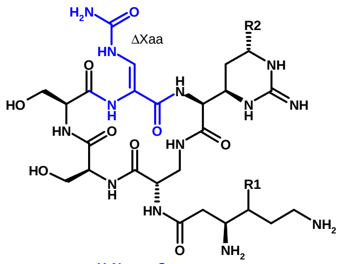

44 capreomycins

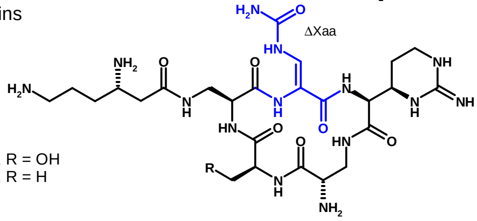

capreomycin IA, R = OH
capreomycin IB, R = H

45 callynormine A
callyaerins A-H

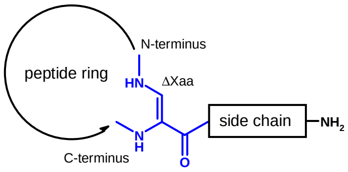

	main chain (ring)								side chain				
	R1	R2	R3	R4	R5	R6	R7	R8	C1	C2	C3	C4	C5
Callynormine A	Ile	Hyp	Val	Leu	Pro	Pro	Leu		Pro	Phe	Leu		
Callyaerins													
A	Ile	Hyp	Val	Ile	Leu	Pro	Pro	Leu	Pro	Ile	Phe	Gly	
B	Ile	Hyp	Ile	Ile	Leu	Pro	Pro	Leu	Pro	Ile	Ile		
C	His	Hyp	Leu	Leu	Pro	Pro	Val		Pro	Leu	Phe	Gly	
D	Ile	Ile	Phe	Pro	Hyp	Pro	Leu		Pro	Ile	Asn	Ala	Ile
E	Leu	Pro	Phe	Phe	Pro	Pro	Val		Pro	Ile	Ile	Gly	
F	Val	Pro	Val	Phe	Pro				Pro	Leu	Phe	Ile	
G	Leu	Pro	Pro	Pro	Pro	Leu			Pro	Phe	Phe	Phe	
H	Val	Pro	Val	Phe	Pro	Pro	Leu		Pro	Ile			
All the L form. Hyp – 3-hydroxyproline													

46 celenamides

A, R1 = Leu, R2 = OH
B, R1 = Val, R2 = OH
C, R1 = Leu, R2 = H
E, R1 = H, R2 = OH

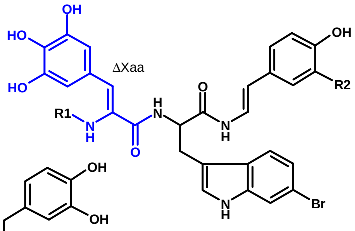

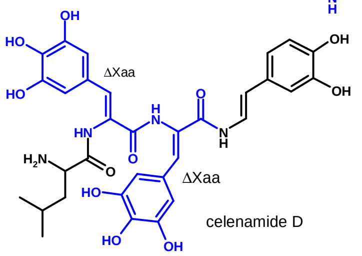

celenamide D

47 tunichromes

An-1 (tunichrome B)
R1 = R2 = OH
An-2, R1 = H, R2 = OH
An-3, R1 = R2 = H

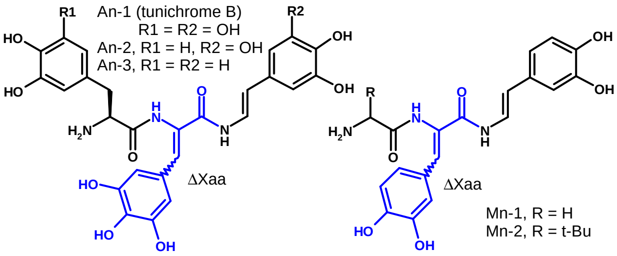

Mn-1, R = H
Mn-2, R = t-Bu

48 azinomycins

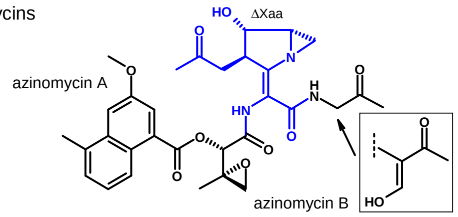

49 dityromycin, GE82832

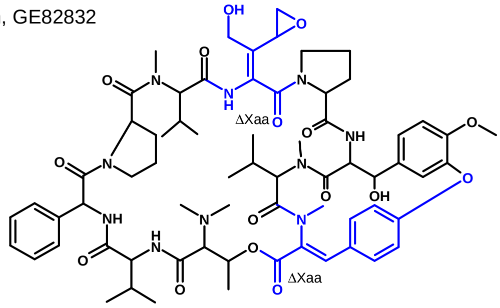

50 microcystin-LA

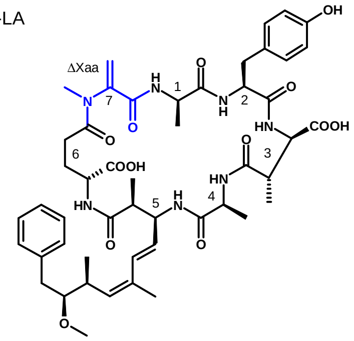

55 mutremdamide A

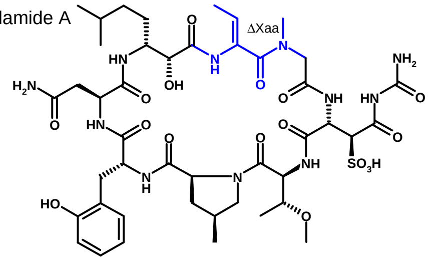

51 FR900359, YM254890-2

FR900359
R1 = $CH(CH_3)_2$
R2 = CH_2CH_3
YM254890
R1 = R2 = CH_3
YM254891
R1 = CH_3
R2 = CH_2CH_3
YM254892
R1 = CH_3, R2 = CH_2SCH_3

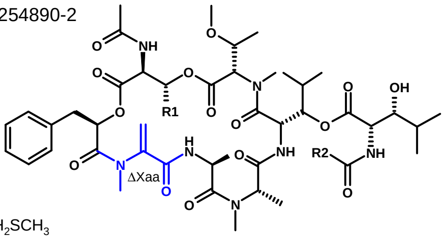

56 cyclothiazomycins

B1 [1 = Arg]
B2 [1 = Arg, 2 = (*E*)-ΔAbu]

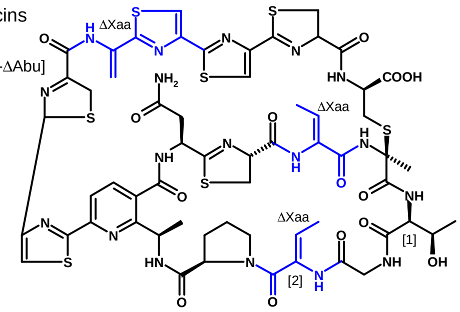

52 BE-22179

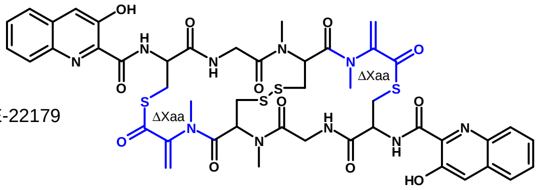

57

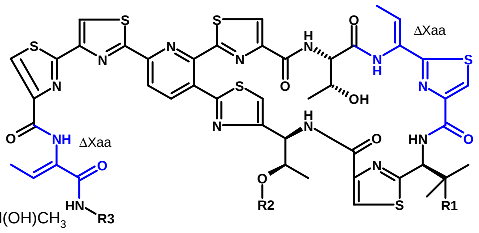

micrococcin P1
R1= R2 =H
R3 = (*R*)-$CH_2CH(OH)CH_3$

micrococcin P2
R1 = R2 = H,
R3 = CH_2COCH_3

thiocillin I
R1=OH, R2 = H
R3 = (*R*)-$CH_2CH(OH)CH_3$

thiocillin II
(unknown stereochemistry)
R1=OH, R2 = CH_3
R3 = $CH_2CH(OH)CH_3$

thiocillin III
(unknown stereochemistry)
R1=H, R2 = CH_3
R3 = $CH_2CH(OH)CH_3$

YM-266183
(unknown stereochemistry)
R1=OH, R2 = H
R3 = CH_2COCH_3

YM-266184
(unknown stereochemistry)
R1=OH, R2 = CH_3
R3 = CH_2COCH_3

53 nodularins

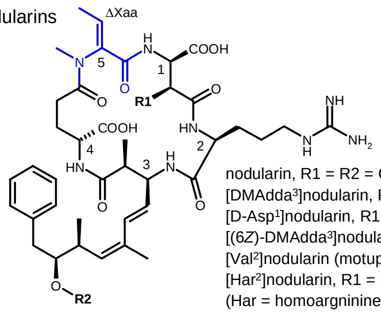

nodularin, R1 = R2 = CH_3
[DMAdda^3]nodularin, R1 = CH_3, R2 = H
[D-Asp^1]nodularin, R1 = H, R2 = CH_3
[(6*Z*)-DMAdda^3]nodularin, R1 = R2 = CH_3
[Val^2]nodularin (motuporin) R1 = R2 = CH_3
[Har^2]nodularin, R1 = R2 = CH_3
(Har = homoargninine)

54 tentoxin

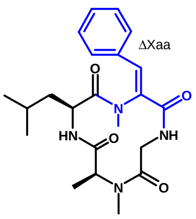

58 nosiheptide

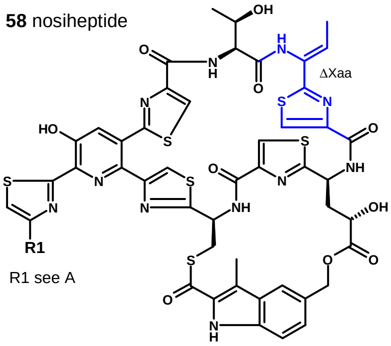

R1 see A

59

	R1	R2	R3	R4
nocathiacin I (MJ347-81F4 A)	see A	H	see a	OH
nocathiacin II	see A	H	see a	H
nocathiacin III	see A	H	H	OH
nocathiacin IV	CONH2	H	see a	OH
thiazomycin A	see A	H	see b	OH
thiazomycin B	see A	H	H	H
thiazomycin C	CONH2	H	see b	H
thiazomycin D	see A	H	see c	OH
MJ347-81F4 B	see A	H	see d	OH
glycothiohexide α	CONH2	H	see a	H
O-Methyl--glycothiohexide α	CONH2	CH3	see a	H

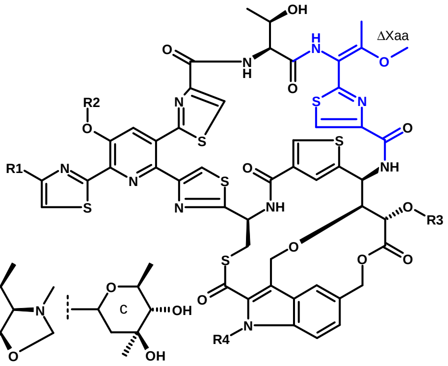

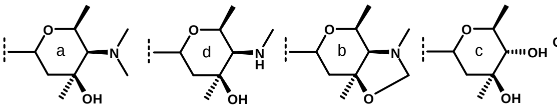

60

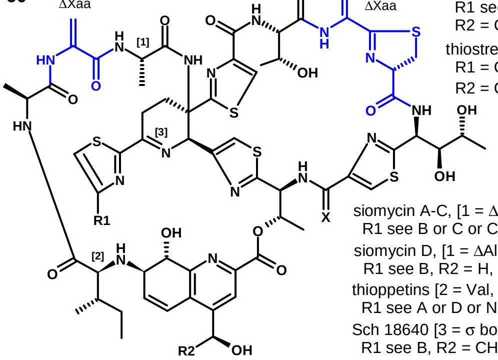

thiostrepton A, R1 see A, R2 = CH_3, x =O
thiostrepton B, R1 = $CONH_2$, R2 = CH_3, x = O

siomycin A-C, [1 = ΔAla, 2 = Val] R1 see B or C or $CONH_2$, R2 = CH_3, x =O
siomycin D, [1 = ΔAla, 2 = Val] R1 see B, R2 = H, x = O
thioppetins [2 = Val, 3 = σ bond (serie B)], R1 see A or D or NH_2,R2 = CH_3, x = S
Sch 18640 [3 = σ bond], R1 see B, R2 = CH_3, x = O

61 Sch40832

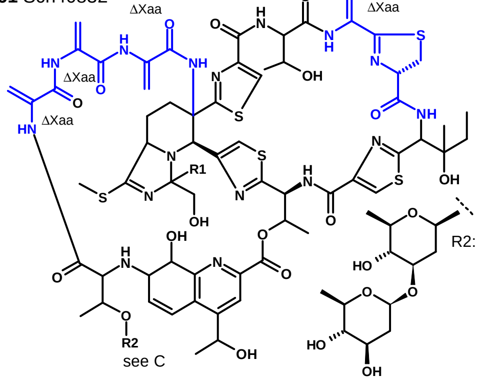

62

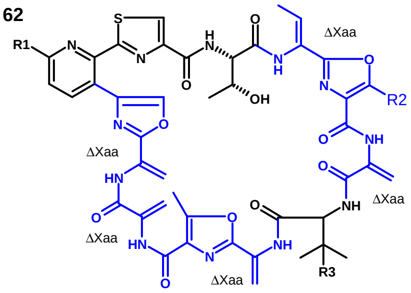

geninthiocin R1 see B, R2 = H,
berninamycin A R1 see B, R2 = CH_3, R3 = OH
berninamycin B R1 see B, R2 = CH_3, R3 = H
berninamycin C R1 see A, R2 = CH_3, R3 = OH
berninamycin D R1 = NH_2, R2 = CH_3, R3 = OH

64

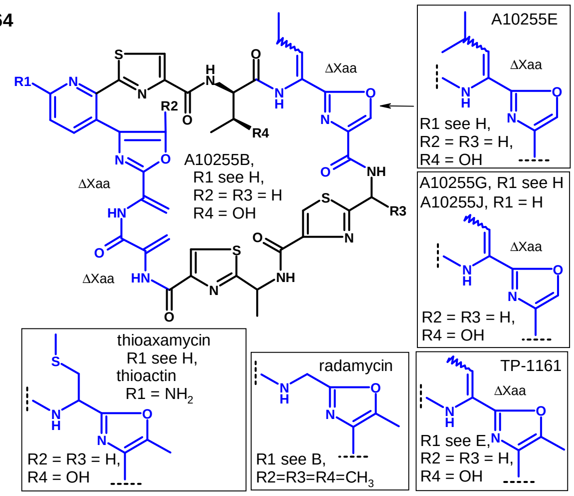

63

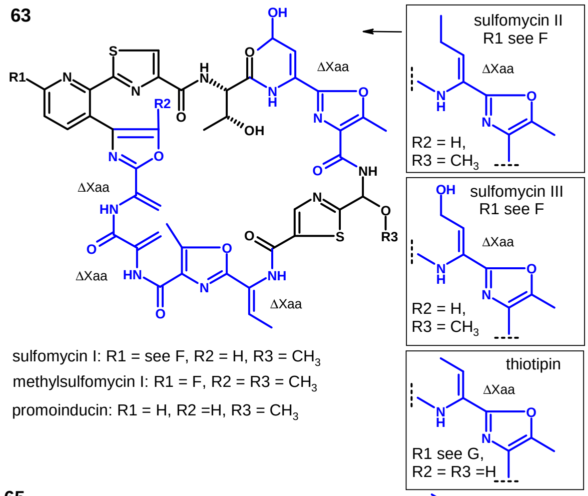

sulfomycin I: R1 = see F, R2 = H, R3 = CH_3
methylsulfomycin I: R1 = F, R2 = R3 = CH_3
promoinducin: R1 = H, R2 =H, R3 = CH_3

66

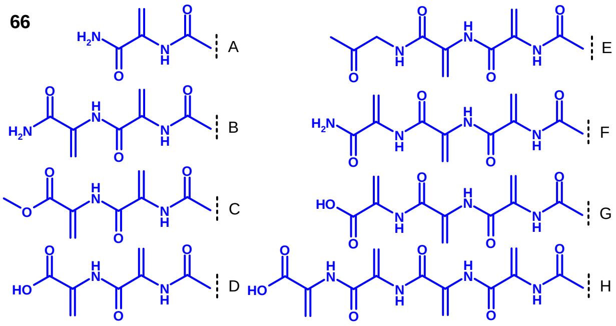

65

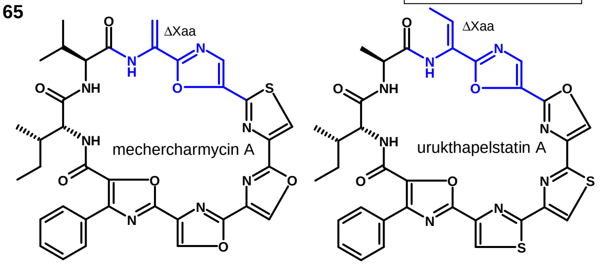

Supplement: Supplementary file 1 — Supplementary material 1 (PDF 160 kb) [file 726_2014_1846_MOESM1_ESM.pdf]
